# Neurosurgical Admission Later Than 4 h After the Emergency Call Does Not Result in Worse Long-Term Outcome in Subarachnoid Haemorrhage

**DOI:** 10.3389/fneur.2021.739020

**Published:** 2021-10-28

**Authors:** Asger Sonne, Jesper B. Andersen, Vagn Eskesen, Freddy Lippert, Frans B. Waldorff, Volkert Siersma, Nicolai Lohse, Lars S. Rasmussen

**Affiliations:** ^1^Department of Anaesthesia, Centre of Head and Orthopaedics, Copenhagen University Hospital Rigshospitalet, Copenhagen, Denmark; ^2^Department of Neurosurgery, The Neuroscience Centre, Copenhagen University Hospital Rigshospitalet, Copenhagen, Denmark; ^3^Department of Clinical Medicine, Faculty of Health and Medical Sciences, University of Copenhagen, Copenhagen, Denmark; ^4^Copenhagen Emergency Medical Services, Copenhagen, Denmark; ^5^Research Unit of General Practice, Department of Public Health, University of Southern Denmark, Odense, Denmark; ^6^The Research Unit for General Practice and Section of General Practice, Department of Public Health, University of Copenhagen, Copenhagen, Denmark; ^7^Department of Emergency Medicine, Copenhagen University Hospital Nordsjællands Hospital, Hillerød, Denmark

**Keywords:** subarachnoid haemorrhage, emergency medical systems, outcome, return-to-work, mortality, long-term outcome, time to admission

## Abstract

**Background:** Few studies have investigated the importance of the time interval between contact to the emergency medical service and neurosurgical admission in patients with spontaneous subarachnoid haemorrhage. We hypothesised that longer time to treatment would be associated with an increased risk of death or early retirement.

**Methods:** This was a retrospective observational study with 4 years follow-up. Those who reached a neurosurgical department in fewer than 4 h were compared with those who reached it in more than 4 h. Individual level data were merged from the Danish National Patient Register, medical records, the Copenhagen Emergency Medical Dispatch Centre, the Civil Registration System, and the Ministry of Employment and Statistics Denmark. Patients were ≥18 years and had a verified diagnosis of spontaneous subarachnoid haemorrhage. The primary outcome was death or early retirement after 4 years.

**Results:** Two hundred sixty-two patients admitted within a three-and-a-half-year time period were identified. Data were available in 124 patients, and 61 of them were in their working age. Four-year all-cause mortality was 25.8%. No significant association was found between time to neurosurgical admission and risk of death or early retirement (OR = 0.35, 95% confidence interval [CI]: 0.10–1.23, *p* = 0.10).

**Conclusion:** We did not find an association between the time from emergency telephone call to neurosurgical admission and the risk of death or early retirement.

## Introduction

Despite accounting for only 5% of strokes, spontaneous subarachnoid haemorrhage (SAH) accounts for 27% of all stroke-related life years lost before the age of 65 (1). It is estimated that 12% die before hospital admission (2), 14% die during admission (3), and after 6 months, 1 in 5 patients, that were admitted in good neurological condition, will be either dead or severely disabled (4, 5).

For the survivors who were employed before their SAH, the proportion regaining functional independence and returning to full-time employment is in the range of 34–67% (6–8). Many of those who do return to work will work fewer hours or hold a position with less responsibility (6).

It is relevant to the early management of SAH patients that poor neurologic outcome is related to poor neurological condition at the time of neurosurgical admission (7). The neurological condition can deteriorate as a result of rebleeding which occurs at a median of 3.5 h after the initial haemorrhage (9).

Some studies have reported a benefit of rapid admission to a neurosurgical department (10, 11), while others have found that delay does not affect outcome (12). Unfortunately, the studies are very heterogeneous which makes comparisons difficult. Furthermore, most studies did not focus on the time interval from the initial healthcare contact to specialised care.

The primary aim of this study was to investigate if there was an association between the time interval from telephone contact with the emergency medical dispatch centre (EMDC) to neurosurgical admission (hereafter: time to treatment), and the risk of death or early retirement during a 4-year follow-up period after SAH. Second, we aimed to investigate if time to treatment was associated with time spent on social welfare and the degree to which survivors return to work. We hypothesised that longer time to treatment would be associated with an increased risk of death or early retirement.

## Methods

### Design and Setting

This was a retrospective cohort study with 4 years follow-up conducted in the Capital Region of Denmark. The region has one hospital with neurosurgical facilities and nine referring hospitals serving approximately 1.8 million people. Medical emergency calls are handled at the Copenhagen Emergency Medical Dispatch Centre. Here, medically trained dispatchers interview the callers and activate appropriate prehospital responses. The urgency of the response is decided by the dispatcher supported by an electronic decision support system (13).

### Cohort

The cohort is described elsewhere (14). From the Danish National Patient Register we extracted patients who were at least 18 years of age, initially admitted to any hospital within the Capital Region of Denmark, and diagnosed with a first-ever SAH (International Classification of Diseases codes I60.0–I60.9). Admissions took place between May 1, 2011, and December 31, 2014. Diagnoses were validated by two independent medical record reviews. SAHs of both aneurysmal and non-aneurysmal non-traumatic origin were included. Both patients directly admitted and secondarily transferred to the neurosurgical department and patients who remained at referring hospitals were included.

### Data Sources

All Danish residents are assigned a unique civil registration number. This number is used as identification throughout the public sector and in all healthcare registries (15). These were used to link individual level data across databases.

#### Medical Records

For patients identified in the Danish National Patient Register, medical records were screened to identify the time of neurosurgical admission, aetiology of the SAH, and date of the potential contact to the EMDC.

#### EMDC

The time of emergency telephone calls and the urgency of the activated prehospital responses were extracted.

#### The Danish Register for Evaluation and Marginalisation

This database is generated by the Ministry of Employment (16). It contains information on all income sources and social transfer payments since 1991. Ninety-five percent of the Danish population appears in the database. Also, retirement in any form is registered. The database is updated on a weekly basis. Data on social transfer payments were extracted from 3 weeks before admission until 4 years after.

Early retirement was defined as a situation where the patient received post-employment benefits, post-employment benefits after employment with reduced work capacity, or early old-age pension.

Social welfare payments were defined as unemployment benefits; cash benefits; educational aid; labour market benefits; rehabilitation aid or being in a protected job; job training; a subsidised job or in a job readiness evaluation process.

#### Statistics Denmark

Information on labour market affiliation was extracted from Statistics Denmark (16). Based on main income sources, each citizen is assigned to 1 of 22 different socioeconomic groups. We extracted patients' socioeconomic level in the calendar year prior to being admitted with SAH. The 22 groups were classified as low, medium, and high income, respectively.

#### Civil Registration System

This national register is updated daily with regards to vital status and emigration. Data are virtually complete and highly accurate (15). For SAH patients, vital status and death dates were extracted.

### Analyses

For descriptive statistics, continuous data were reported as medians with inter-quartile range (IQR) and proportions with 95% confidence intervals (CI). Continuous data were compared by the Mann-Whitney U-test and proportions by the χ^2^-test.

Death by any cause within 4 years was computed for all SAH patients.

Patients aged 18–60 years and employed (any number of hours) 3 weeks before admission were classified as *available to the labour market*. Sixty years of age was chosen as the upper limit for working age as reduced work capacity may more often result in early voluntary retirement in patients older than this. For those available to the labour market before admission, we calculated the proportion of patients on involuntary early retirement after 4 years, the proportion of time on social welfare during the 4 years of follow-up, and the number of weekly working hours before and after their SAH.

For the primary and secondary analyses, we included those available to the labour market at the time of admission, and for whom information on the time of contact to the EMDC was known. We used multivariable logistic regression to assess the association between time to treatment and the risk of death or involuntary early retirement within 4 years. For survivors who had not retired early, we assessed the association between time to treatment and the risk of spending more than 50% of the first 4 years on social transfer payments, as well as the risk of working fewer hours 4 years after SAH as compared to before.

The median time from initial haemorrhage to early rebleeding has been reported to be 3.5–4 h (9, 17). Time to treatment was dichotomized around 4 hours (referred to as short and long time to treatment, respectively). If a patient was deemed moribund and remained at a referring hospital, the time of neurosurgical consultation was used as the time of arrival to the neurosurgical department. Analyses were adjusted for the potential confounders: year (2011, 2012, 2013, or 2014), age (≤ 49 or ≥50 years), sex, socioeconomic status before admission (high, middle, or low), and the level of urgency. The latter was categorised according to the type of prehospital resource being dispatched (ambulance with/without lights and sirens or referral to out-of-hours general practitioner). Results were reported as adjusted odds ratios (OR) with 95% CI.

Estimating a 60% risk of death or early retirement in the group with the longest time to neurosurgical admission and a 30% risk in the other group, we calculated that 80 patients would provide a 76% power at the 0.05 significance level to detect that difference.

Statistical analyses were performed in SAS Enterprise Guide v.9.4. We considered *p* < 0.05 as statistically significant.

### Legal Aspects

The study was approved by the Danish Data Protection Agency and the Danish Health and Medicines Authority. Approval by the Danish Committee System on Health Research Ethics was waived.

### Reporting Guideline

Reporting was done according to the STROBE guideline.

### Study Registration

NCT04377347 (www.clinicaltrials.gov).

## Results

**Figure 1:**
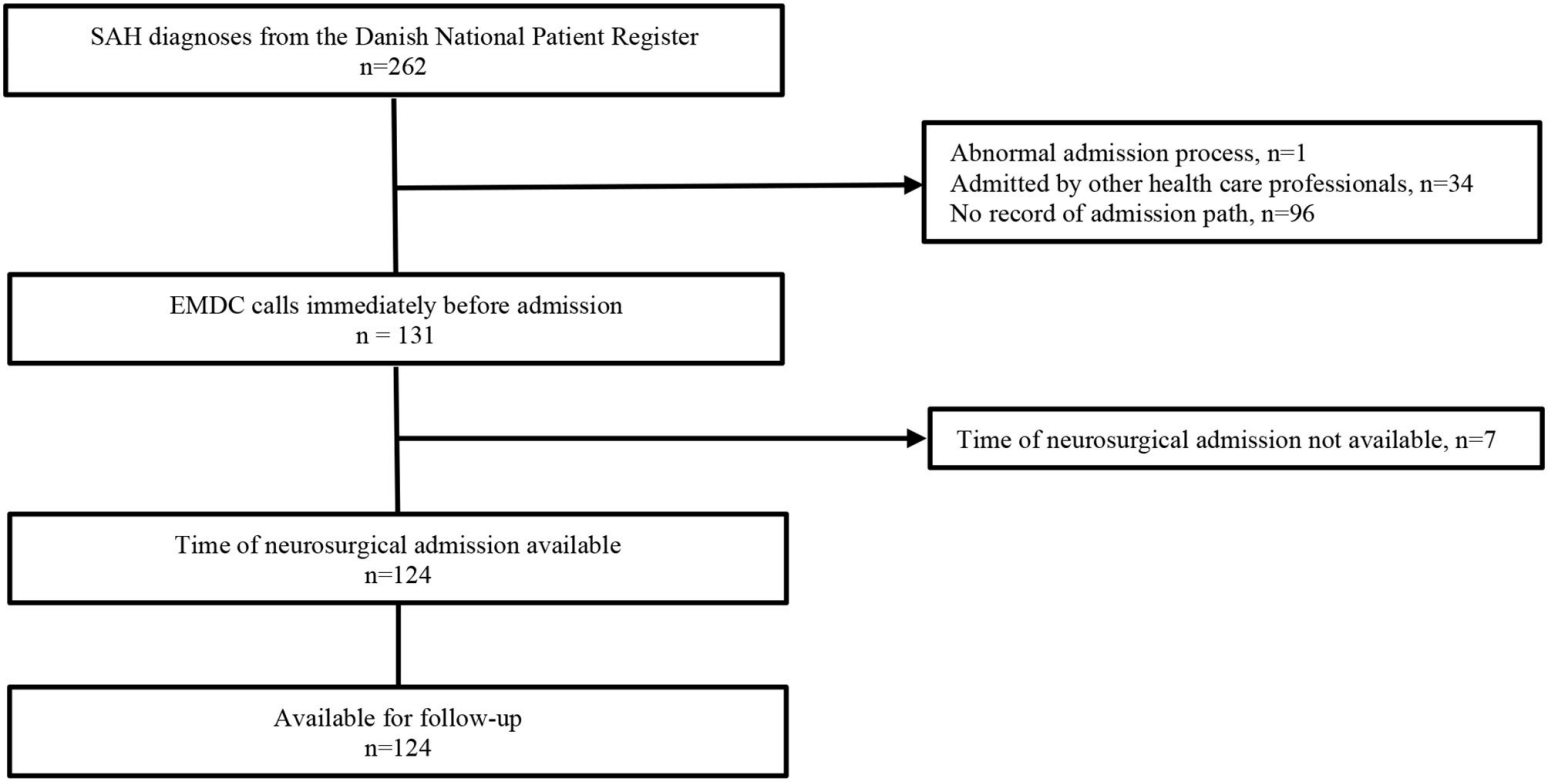
Patient inclusion from original cohort. SAH, spontaneous subarachnoid haemorrhage; n, number; EMDC, emergency medical dispatch centre.

Two hundred sixty-two SAH patients were admitted within the study period. The EMDC was contacted by the patient or a bystander in 131 of these cases and complete data were available in 124 patients (Figure 1). Median age was 59.0 (IQR: 48–70) and 57.0 (IQR: 48.0–66.0) among those who contacted EMDC and those who did not, respectively (*p* = 0.28). Females made up 67.2% (95% CI: 59.1–75.2) and 61.8% (95% CI: 53.5–70.2), respectively. Of the 131 who did not call the EMDC, 22 had died after 4 years (16.8%, 95% CI: 10.4–23.2), while 35 of the 131 who called the EMDC had died (26.7%, 19.1–34.3) (*p* = 0.05).

Of the 124 patients included, none were lost to follow-up. Among these the 4-year mortality was 25.8% (95% CI: 18.1–33.5) (Table 1).

**Table 1:** Time to treatment and long-term outcome in spontaneous subarachnoid haemorrhage.

	**Time to treatment (all patients)**						**Time to treatment (available to labour market before SAH)**					
	**Total**	**Short**	**Long**		**Long vs. short**		**Total**	**Short**	**Long**		**Long vs. short**	
	***n =* 124 (%)**	***n =* 78 (%)**	***n =* 46 (%)**	***P***	**OR (95%CI)**	***P***	***n =* 61 (%)**	***n =* 38 (%)**	***n =* 23 (%)**	***P***	**OR (95%CI)**	***P***
Year				0.11						0.65		
'2011	25 (20.2)	16 (64.0)	9 (36.0)				15 (24.6)	11 (73.3)	4 (26.7)			
'2012	36 (29.0)	17 (47.2)	19 (52.8)				17 (27.9)	9 (52.9)	8 (47.1)			
'2013	39 (31.5)	27 (69.2)	12 (30.8)				20 (32.9)	13 (65.0)	7 (35.0)			
'2014	24 (19.4)	18 (75.0)	6 (25.0)				9 (14.8)	5 (55.6)	4 (44.4)			
Age				0.95						0.33		
≤ 49 years	37 (29.8)	24 (64.9)	13 (35.1)				34 (55.7)	23 (67.7)	11 (32.4)			
50–69 years	56 (45.2)	35 (62.5)	21 (37.5)				27 (44.3)	15 (55.6)	12 (44.4)			
≥70 years	31 (25.0)	19 (61.3)	12 (38.7)				-	-	-			
Sex				0.42						0.87		
Woman	81 (65.3)	53 (65.4)	28 (34.6)				39 (63.9)	24 (61.5)	15 (38.5)			
Man	43 (34.7)	25 (58.1)	18 (41.9)				22 (36.1)	14 (63.6)	8 (36.4)			
Income				0.93						0.89		
High	16 (12.9)	10 (62.5)	6 (37.5)				16 (26.2)	10 (62.5)	6 (37.5)			
Middle	43 (34.7)	28 (65.1)	15 (34.9)				36 (59.0)	23 (63.9)	13 (36.1)			
Low	65 (52.4)	40 (61.5)	25 (38.5)				9 (14.8)	5 (55.6)	4 (44.4)			
Perimesencephalic haemorrhage				0.99						-		
No	122 (98.4)	77 (63.1)	45 (36.9)				61 (100.0)	38 (62.3)	23 (37.7)			
Yes	2 (1.6)	1 (50.0)	1 (50.0)				0 (0.0)	0 (0.0)	0 (0.0)			
Emergency medical dispatch response				0.01						0.03		
Ambulance with lights/sirens	95 (77.9)	67 (70.5)	28 (29.5)				43 (71.7)	31 (72.1)	12 (27.9)			
Ambulance without lights/sirens	24 (19.7)	9 (37.5)	15 (62.5)				17 (28.3)	7 (41.2)	10 (58.8)			
Referred to out-of-hours GP	3 (2.4)	2 (66.7)	1 (33.3)				0 (0.0)	0 (0.0)	0 (0.0)			
Available to the labour market before SAH				0.89							
No	63 (50.8)	40 (63.5)	23 (36.5)									
Yes	61 (49.2)	38 (62.3)	23 (37.7)									
Death within 4 years after SAH				0.10						0.07		
No	92 (74.2)	54 (58.7)	38 (41.3)				51 (83.6)	29 (56.9)	22 (43.1)			
Yes	32 (25.8)	24 (75.0)	8 (25.0)		0.43 (0.15–1.20)	0.11	10 (16.4)	9 (90.0)	1 (10.0)		0.03 (0.00–1.09)	0.06
Death or early retirement within 4 years after SAH										0.11		
No							36 (59.0)	19 (52.8)	17 (47.2)			
Yes							25 (41.0)	19 (76.0)	6 (24.0)		0.35 (0.10–1.23)	0.10
Working less than full time before SAH										0.57		
No							45 (73.8)	29 (64.4)	16 (35.6)			
Yes							16 (26.2)	9 (56.3)	7 (43.7)			
Reduced labour market affiliation 4 years after SAH										0.60		
No							24 (39.3)	16 (66.7)	8 (33.3)			
Yes (includes death and early pension)							37 (60.7)	22 (59.5)	15 (40.5)		1.52 (0.42–5.46)	0.52
							**Time to treatment (alive and not on early retirement)**					
							**Total**	**Short**	**Long**		**Long vs. short**	
							***n =*** **36 (%)**	***n =*** **19 (%)**	***n =*** **17 (%)**	**P**	**OR (95%CI)**	**P**
Employed but working fewer hours than before SAH										0.03		
No							24 (66.7)	16 (66.7)	8 (33.3)			
Yes							12 (33.3)	3 (25.0)	9 (75.0)		9.87 (1.33–73.10)	0.03
Employed but on social welfare ≥50% of the 4 years after SAH										0.16		
No							24 (66.7)	15 (62.5)	9 (37.5)			
Yes							12 (33.3)	4 (33.3)	8 (66.7)		6.70 (0.77–58.38)	0.09

*Multivariable logistic regression analysis of time to treatment and long-term outcomes after spontaneous subarachnoid haemorrhage (SAH). n, number; SAH, spontaneous subarachnoid haemorrhage; OR, Odds ratio; GP, general practitioner*.

The median time to treatment was 207.5 (IQR 147.0–304.5) min and 78 patients (62.9%, 95% CI: 54.4–71.4) reached the neurosurgical department in 4 hours or less. Thirty-three (27.7%, 95% CI: 19.7–35.8) patients were directly admitted to a hospital with neurosurgical facilities while 4 (3.4%, 95% CI: 0.1–6.6) patients remained at the referring hospitals after neurosurgical consultation. All four were older than 70 years. In 5 patients, data for the first admission were unavailable. Ninety-five (77.9%, 95% CI: 70.5–85.2) patients received an ambulance with lights and sirens. In 2 patients, data on the dispatched response were unavailable.

Of the 124 patients included, 61 (49.2%, 95% CI: 40.4–58.0) were available to the labour market before SAH. Twenty-five (41.0%, 95% CI: 28.6–53.3) of these were either dead or had retired early 4 years after their SAH. Among the 36 (59.0%, 95% CI: 46.7–71.4) survivors who had not retired after 4 years, 12 (33.3%, 95% CI: 17.9–48.7) had been on social transfer payment for more than 50% of the 4-year follow-up period. After four years 12 (33.3%, 95% CI: 17.9–48.7) worked fewer hours than before their SAH. Patients with longer time to treatment tended to have a lower risk of death or early retirement after 4 years (OR = 0.35, 95% CI: 0.10–1.23, *p* = 0.10) but tended to have a higher risk of being on social welfare for more than 50% of the time (OR = 6.70, 95% CI: 0.77–58.38, *p* = 0.09). Patients with longer time to treatment, and who were not dead or had retired after 4 years, had a significantly higher risk of working fewer hours (OR = 9.87, 95% CI: 1.33–73.10, *p* = 0.03).

None of the patients available to the labour market had SAH caused by a perimesencephalic haemorrhage.

## Discussion

The OR for the association between time to treatment and the risk of death or early retirement was 0.35. This suggests that a short time interval was not beneficial. However, the opposite was found for the risk of being on social welfare for more than 50% of the time and working fewer hours, but the confidence intervals were wide.

Patients with short time to treatment tended to have worse outcome and this may reflect that patients with the most severe symptoms are triaged faster to a hospital with neurosurgery. However, among patients with less severe haemorrhages, longer time to treatment seemed to be disadvantageous.

Surprisingly, only 47% of SAH patient had been admitted via the EMDC. These patients had a higher mortality, and this could indicate that the EMDC was primarily contacted when more severe haemorrhages occurred.

Despite having higher mortality than the non-EMDC patients, the four-year mortality of EMDC patients was low compared to the median 43% reported in European studies, but mortality reports vary widely (18). Including those who died, 60.7% (*n* = 37) of patients aged 18–60 years had reduced labour market affiliation 4 years after SAH in our study. Among those who returned to work, 66.7% worked to the same extent as before. This is comparable to the 75% reported in a study of long-term outcomes restricted to patients that were in a good neurological condition at discharge (19). Our study was based on data from an area with easy access to emergency medical services, short response times, a highly specialised neurosurgical and neurointensive care departments, extensive rehabilitation services, and free access to health care for all residents. The good outcome observed in many patients in this study most likely reflects a combination of these factors more than any single element being performed optimally.

Being able to return to work requires good neurological and psychological recovery which only 25% of survivors report (20). Long-term neurological and psychological problems, in return, are associated with poor neurological condition at admission (21). Eighty-three percent of Hunt-&-Hess grade I patients return to work while only 43% of Hunt-&-Hess grade III patients return (7). Along with the early brain injury caused by the initial SAH, rebleeding is the most important cause of early neurological deterioration and 50% of these occur within the first 3.5 h (9). Both a recent systematic review on delays in SAH management (22) and the Utstein recommendation for emergency stroke care (23) emphasise that minimising time to specialist treatment is important. However, the optimal timing of admission and type of receiving hospital is still debated, as some find shorter time to treatment beneficial (11), while others argue that initial admission to the closest hospital for resuscitation and stabilisation prior to interhospital transfer should be prioritised (24). Prolonged transfer time in itself has not been found to result in higher mortality or worse neurological outcome (25) but prolonged transfer time increases the risk of aneurysm rerupture (26). A Norwegian study reported an overall risk of rebleeding of 0.8% for every hour from symptom onset to neurosurgical admission. In Hunt-&-Hess grade V patients, the rebleeding rate was 6.5% per hour (25).

Transferring the patient to the neurosurgical department constitutes 21% of the total time spent from symptom onset to final treatment (22). Patients surviving (27) and returning to work are known to be in a better clinical condition on admission (28) and it seems logical that we should minimise the time spent in the prehospital phase.

Prior research on the timing of neurosurgical admission has rarely focused on the time spent immediately after patients' first contact with the emergency medical services. With an excess mortality among those admitted fast, our results indicate that the most severe cases of SAH are effectively triaged to highly specialised care early. A similar pattern was seen by Sorteberg et al., who reported a mortality of 36.8% in patients admitted directly to a neurosurgical department vs. 18.6% in whose who were secondarily transferred. Our results also give notion to the idea that patients who experience less severe SAH (i.e., those who can return to work) may be prone to delayed neurosurgical admission and that this delay may be associated with reduced work ability. Even among those with less severe SAH the timing of neurosurgical admission may thus be of great importance for patients' outcome. Previous studies on patients with less severe SAHs have used mortality and rebleeding as their outcome measures (24, 25), but using return-to-work as an outcome measure we have found the timing of neurosurgical admission to be important also in this group of patients. Contrary to what previous studies have concluded (24, 25), our results suggest that also less severely affected patients benefit from early neurosurgical admission.

The main strength of this study was that we were able to follow our entire cohort for 4 years with a high degree of detail through high-quality databases with prospectively registered data. This meant that our study was free from recall bias. Second, the diagnoses were validated by medical record review, and finally we focused on a large well-defined geographical region with all emergency telephone calls coming through only one EMDC.

The study had limitations as well. There are currently no published guidelines defining the optimal timing of neurosurgical care. Based on current evidence of rebleeding, and clinical experience, we therefore defined a clinically relevant cut-off as being 4 h. The lack of data on haemorrhage severity is another limitation. No scoring systems have been developed to quantify SAH severity in telephone triage. The urgency of the dispatched ambulance was used as the best available proxy. We were only able to include those who survived to hospital admission because the diagnosis requires a brain scan. We included all eligible patients from the original cohort; however, the number of patients included in the analyses does not provide much statistical power to detect a clinically important association between time to treatment and outcome. The latter is caused by missing data in more than half of the cohort, which in itself is also a limitation.

## Conclusion

We found a four-year all-cause mortality of 25.8% in patients with spontaneous subarachnoid haemorrhage who called the EMDC. Admission to a neurosurgical department more than 4 h after contacting the EMDC was not found to be significantly associated with an increased risk of death or early retirement or to time on social welfare. In survivors who returned to work, neurosurgical admission after more than 4 h was associated with an increased risk of working fewer hours than before the SAH.

## Data Availability Statement

The data analysed in this study is subject to the following licences/restrictions: Datasets are the property of individual databases and only accessible with specific authorizations. Requests to access these datasets should be directed to Danmarks Statistik, www.dst.dk. Copenhagen Emergency Medical Services, www.regionh.dk.

## Author Contributions

AS: study design, data collection, and drafting manuscript. JA: data collection and revision of manuscript. VE, FL, FW, NL, and LR: study design, analysis plan, and revision of manuscript. VS: analysis plan, performing analyses, and revision of manuscript. All authors contributed to the article and approved the submitted version.

## Funding

Funding was received by Trygfonden, Grant No. 121964.

## Conflict of Interest

FL reports grants from Trygfonden and Laerdalfonden outside the submitted work. The remaining authors declare that the research was conducted in the absence of any commercial or financial relationships that could be construed as a potential conflict of interest.

## Publisher's Note

All claims expressed in this article are solely those of the authors and do not necessarily represent those of their affiliated organizations, or those of the publisher, the editors and the reviewers. Any product that may be evaluated in this article, or claim that may be made by its manufacturer, is not guaranteed or endorsed by the publisher.

## Abbreviations

SAH: Spontaneous subarachnoid haemorrhage
EMDC: Emergency Medical Dispatch Centre.
